# 2-Hydr­oxy-10-propargylpyrrolo[2,1-*c*][1,4]benzodiazepine-5,11-dione monohydrate

**DOI:** 10.1107/S1600536810006896

**Published:** 2010-03-03

**Authors:** S. Ourahou, M. Chammache, H. Zouihri, El Mokhtar Essassi, Seik Weng Ng

**Affiliations:** a Laboratoire de Chimie Organique Hétérocyclique, Pôle de Compétences Pharmacochimie, Université Mohammed V-Agdal, BP 1014 Avenue Ibn Batout, Rabat, Morocco; b CNRST, Division of UATRS Angle Allal Fassi/FAR, BP 8027 Hay Riad, 10000 Rabat, Morocco; c Department of Chemistry, University of Malaya, 50603 Kuala Lumpur, Malaysia

## Abstract

The title compound, C_15_H_14_N_2_O_3_·H_2_O, consists of a benzodiazepinedione system fused to a pyrrole system. The seven-membered ring adopts a boat-shaped conformation (with the methine C atom as the prow); the five-membered ring adopts an enveloped-shaped conformation (with the hydr­oxy-bearing C atom as the flap). In the crystal, adjacent mol­ecules are linked by O—H⋯O hydrogen bonds into sheets parallel to (102). In addition, C_acetyl­inic_—H⋯O hydrogen bonds occur.

## Related literature

Pyrrolo[2,1-*c*][1,4]benzodiazepines are potent anti­biotics produced by *Streptomyces *species; see: Cargill *et al.* (1974 ▶). For the design of DNA inter-strand cross-linking and conjugate agents to enhance the sequence selectivity and selectivity for tumor cells, see: Gregson *et al.* (2004 ▶).
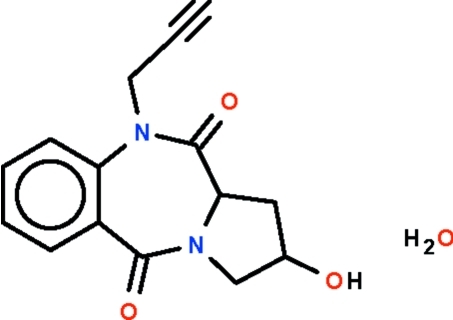


## Experimental

### 

#### Crystal data

C_15_H_14_N_2_O_3_·H_2_O
*M* *_r_* = 288.30
Monoclinic, 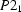

*a* = 6.8977 (1) Å
*b* = 7.9761 (1) Å
*c* = 13.0680 (2) Å
β = 99.194 (1)°
*V* = 709.72 (2) Å^3^
*Z* = 2
Mo *K*α radiation
μ = 0.10 mm^−1^
*T* = 293 K
0.3 × 0.3 × 0.3 mm

#### Data collection

Bruker APEXII diffractometer
9524 measured reflections
1744 independent reflections
1680 reflections with *I* > 2σ(*I*)
*R*_int_ = 0.021

#### Refinement

*R*[*F* ^2^ > 2σ(*F* ^2^)] = 0.029
*wR*(*F* ^2^) = 0.094
*S* = 1.10
1744 reflections
202 parameters
4 restraints
H atoms treated by a mixture of independent and constrained refinement
Δρ_max_ = 0.18 e Å^−3^
Δρ_min_ = −0.16 e Å^−3^

### 

Data collection: *APEX2* (Bruker, 2005 ▶); cell refinement: *SAINT* (Bruker, 2005 ▶); data reduction: *SAINT*; program(s) used to solve structure: *SHELXS97* (Sheldrick, 2008 ▶); program(s) used to refine structure: *SHELXL97* (Sheldrick, 2008 ▶); molecular graphics: *X-SEED* (Barbour, 2001 ▶); software used to prepare material for publication: *publCIF* (Westrip, 2010 ▶).

## Supplementary Material

Crystal structure: contains datablocks global, I. DOI: 10.1107/S1600536810006896/bt5199sup1.cif

Structure factors: contains datablocks I. DOI: 10.1107/S1600536810006896/bt5199Isup2.hkl

Additional supplementary materials:  crystallographic information; 3D view; checkCIF report

## Figures and Tables

**Table 1:** Hydrogen-bond geometry (Å, °)

*D*—H⋯*A*	*D*—H	H⋯*A*	*D*⋯*A*	*D*—H⋯*A*
O3—H3⋯O1w	0.84 (1)	1.85 (1)	2.686 (2)	177 (3)
O1w—H11⋯O2^i^	0.84 (1)	1.92 (1)	2.7485 (19)	172 (4)
O1w—H12⋯O3^ii^	0.84 (1)	1.92 (1)	2.767 (2)	177 (3)
C15—H15⋯O1^iii^	0.93	2.29	3.166 (3)	157

Symmetry codes: (i) 
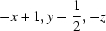
; (ii) 
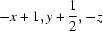
; (iii) 
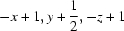
.

**Fig. 1.**
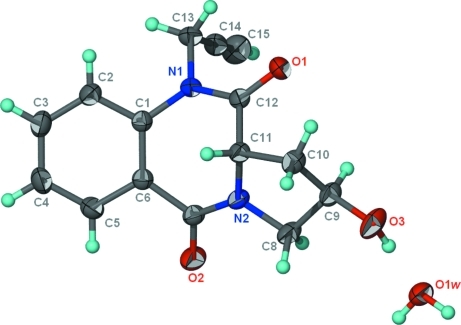
Anisotropic displacement ellipsoid plot (Barbour, 2001) of C15H14N2O3.H2O at the 50% probability level; hydrogen atoms are drawn as spheres of an arbitrary radius.

## supplementary crystallographic information

### Experimental

2-Hydroxy-pyrrolo[2,1-*c*][1,4]benzodiazepine-5,11-dione (0.5 g, 2.15 mmol), propargyl bromide (0.26 g, 2.15 mmol) and potassium carbonate (0.6 g, 4.3 mmol) along with a catalytic amount of tetra-*n*-butyl ammonium bromide were stirred in *N*,*N*-dimethylformamide (20 ml) for 72 h. The solid material was removed by filtration and the solvent evaporated under vacuum. The residue was separated by chromatography on silica gel with an *n*-hexane:ethyl acetate (1:9) solvent system. The compound was obtained as colorless crystals in 50% yield upon evaporation of the solvent.

### Refinement

Carbon-bound H-atoms were placed in calculated positions (C—H 0.93-0.98 Å) and were included in the refinement in the riding model approximation, with *U*(H) set to 1.2*U*(C). The oxygen-bound H-atoms were located in a difference Fourier map, and were refined isotropically with a distance restraint of O–H 0.84±0.01 Å. Due to the absence of anomalous scatterers Friedel pairs were merged and the absolute configuration was arbitrarily set.

### Crystal data

**Table:**

C_15_H_14_N_2_O_3_·H_2_O	*F*(000) = 304
*M**_r_* = 288.30	*D*_x_ = 1.349 Mg m^−^^3^
Monoclinic, *P*2_1_	Mo *K*α radiation, λ = 0.71073 Å
Hall symbol: P 2yb	Cell parameters from 6455 reflections
*a* = 6.8977 (1) Å	θ = 2.5–34.3°
*b* = 7.9761 (1) Å	µ = 0.10 mm^−^^1^
*c* = 13.0680 (2) Å	*T* = 293 K
β = 99.194 (1)°	Block, colorless
*V* = 709.72 (2) Å^3^	0.3 × 0.3 × 0.3 mm
*Z* = 2	

### Data collection

**Table:**

Bruker APEXII diffractometer	1680 reflections with *I* > 2σ(*I*)
Radiation source: fine-focus sealed tube	*R*_int_ = 0.021
graphite	θ_max_ = 27.5°, θ_min_ = 1.6°
φ and ω scans	*h* = −8→8
9524 measured reflections	*k* = −10→10
1744 independent reflections	*l* = −16→16

### Refinement

**Table:**

Refinement on *F*^2^	Primary atom site location: structure-invariant direct methods
Least-squares matrix: full	Secondary atom site location: difference Fourier map
*R*[*F*^2^ > 2σ(*F*^2^)] = 0.029	Hydrogen site location: inferred from neighbouring sites
*wR*(*F*^2^) = 0.094	H atoms treated by a mixture of independent and constrained refinement
*S* = 1.10	*w* = 1/[σ^2^(*F*_o_^2^) + (0.0656*P*)^2^ + 0.0541*P*] where *P* = (*F*_o_^2^ + 2*F*_c_^2^)/3
1744 reflections	(Δ/σ)_max_ = 0.001
202 parameters	Δρ_max_ = 0.18 e Å^−^^3^
4 restraints	Δρ_min_ = −0.16 e Å^−^^3^

### Fractional atomic coordinates and isotropic or equivalent isotropic displacement parameters (Å^2^)

**Table:**

	*x*	*y*	*z*	*U*_iso_*/*U*_eq_	
O1	0.2007 (2)	0.5000 (2)	0.37937 (11)	0.0464 (4)	
O2	0.0918 (2)	1.01924 (19)	0.13444 (13)	0.0503 (4)	
O3	0.4060 (2)	0.4426 (2)	0.06723 (14)	0.0561 (4)	
O1W	0.6986 (2)	0.6295 (2)	0.01457 (11)	0.0465 (4)	
N1	−0.0173 (2)	0.7109 (2)	0.37874 (10)	0.0320 (3)	
N2	0.1101 (2)	0.74500 (19)	0.17437 (11)	0.0317 (3)	
C1	−0.1606 (2)	0.8238 (2)	0.32657 (12)	0.0301 (3)	
C2	−0.3329 (2)	0.8493 (3)	0.36900 (14)	0.0377 (4)	
H2	−0.3559	0.7850	0.4252	0.045*	
C3	−0.4683 (3)	0.9684 (3)	0.32844 (16)	0.0456 (5)	
H3A	−0.5816	0.9835	0.3576	0.055*	
C4	−0.4380 (3)	1.0654 (3)	0.24530 (15)	0.0466 (5)	
H4	−0.5281	1.1478	0.2194	0.056*	
C5	−0.2715 (3)	1.0388 (3)	0.20077 (14)	0.0410 (4)	
H5	−0.2512	1.1033	0.1442	0.049*	
C6	−0.1333 (2)	0.9169 (2)	0.23914 (12)	0.0311 (3)	
C7	0.0340 (3)	0.8983 (2)	0.17963 (13)	0.0330 (3)	
C8	0.2643 (3)	0.7095 (2)	0.11252 (15)	0.0392 (4)	
H8A	0.3734	0.7867	0.1290	0.047*	
H8B	0.2141	0.7161	0.0389	0.047*	
C9	0.3253 (3)	0.5312 (2)	0.14444 (15)	0.0380 (4)	
H9	0.4195	0.5329	0.2091	0.046*	
C10	0.1339 (3)	0.4509 (2)	0.16327 (16)	0.0387 (4)	
H10A	0.1590	0.3553	0.2094	0.046*	
H10B	0.0562	0.4146	0.0986	0.046*	
C11	0.0298 (2)	0.5904 (2)	0.21314 (12)	0.0308 (3)	
H11A	−0.1126	0.5841	0.1909	0.037*	
C12	0.0799 (2)	0.5931 (2)	0.33103 (12)	0.0319 (3)	
C13	0.0327 (3)	0.7196 (3)	0.49276 (13)	0.0362 (4)	
H13A	−0.0758	0.7703	0.5204	0.043*	
H13B	0.0502	0.6067	0.5204	0.043*	
C14	0.2105 (3)	0.8159 (3)	0.52714 (13)	0.0402 (4)	
C15	0.3548 (3)	0.8909 (4)	0.55819 (18)	0.0568 (6)	
H15	0.4689	0.9501	0.5827	0.068*	
H3	0.498 (3)	0.499 (4)	0.049 (2)	0.065 (8)*	
H11	0.760 (4)	0.587 (4)	−0.0291 (19)	0.065 (8)*	
H12	0.663 (4)	0.725 (2)	−0.009 (2)	0.058 (8)*	

### Atomic displacement parameters (Å^2^)

**Table:**

	*U*^11^	*U*^22^	*U*^33^	*U*^12^	*U*^13^	*U*^23^
O1	0.0455 (7)	0.0516 (9)	0.0432 (7)	0.0169 (7)	0.0107 (6)	0.0127 (6)
O2	0.0618 (9)	0.0320 (7)	0.0644 (9)	0.0021 (7)	0.0322 (7)	0.0110 (7)
O3	0.0670 (10)	0.0393 (8)	0.0728 (11)	−0.0023 (8)	0.0442 (9)	−0.0149 (8)
O1W	0.0522 (8)	0.0481 (9)	0.0430 (7)	0.0059 (7)	0.0196 (6)	0.0032 (6)
N1	0.0346 (7)	0.0360 (8)	0.0258 (6)	0.0029 (6)	0.0058 (5)	0.0028 (6)
N2	0.0376 (7)	0.0276 (7)	0.0324 (7)	−0.0025 (6)	0.0138 (6)	−0.0006 (5)
C1	0.0315 (7)	0.0298 (7)	0.0288 (7)	0.0000 (6)	0.0046 (6)	−0.0032 (6)
C2	0.0358 (8)	0.0443 (10)	0.0344 (8)	0.0016 (8)	0.0098 (6)	0.0005 (7)
C3	0.0353 (9)	0.0591 (13)	0.0434 (9)	0.0101 (9)	0.0097 (7)	−0.0037 (9)
C4	0.0435 (9)	0.0510 (12)	0.0439 (9)	0.0178 (9)	0.0024 (7)	0.0002 (9)
C5	0.0481 (10)	0.0391 (10)	0.0360 (8)	0.0083 (8)	0.0072 (7)	0.0042 (8)
C6	0.0341 (7)	0.0290 (8)	0.0305 (7)	0.0003 (6)	0.0060 (6)	−0.0019 (6)
C7	0.0377 (8)	0.0304 (8)	0.0324 (7)	−0.0006 (7)	0.0106 (6)	0.0011 (6)
C8	0.0488 (10)	0.0324 (9)	0.0416 (9)	−0.0030 (8)	0.0233 (8)	−0.0029 (7)
C9	0.0418 (9)	0.0337 (9)	0.0419 (9)	0.0012 (7)	0.0169 (7)	−0.0049 (7)
C10	0.0466 (9)	0.0272 (8)	0.0455 (10)	−0.0029 (7)	0.0169 (8)	−0.0051 (7)
C11	0.0339 (7)	0.0262 (7)	0.0334 (7)	−0.0019 (6)	0.0089 (6)	−0.0001 (6)
C12	0.0307 (7)	0.0324 (8)	0.0338 (7)	−0.0001 (7)	0.0086 (6)	0.0043 (7)
C13	0.0412 (9)	0.0408 (9)	0.0265 (7)	0.0014 (8)	0.0054 (6)	0.0031 (7)
C14	0.0420 (9)	0.0468 (11)	0.0313 (7)	0.0063 (8)	0.0040 (7)	0.0016 (8)
C15	0.0472 (10)	0.0728 (16)	0.0483 (11)	−0.0071 (12)	0.0014 (8)	−0.0078 (12)

### Geometric parameters (Å, °)

**Table:**

O1—C12	1.215 (2)	C4—H4	0.9300
O2—C7	1.230 (2)	C5—C6	1.398 (2)
O3—C9	1.416 (2)	C5—H5	0.9300
O3—H3	0.838 (10)	C6—C7	1.498 (2)
O1W—H11	0.835 (10)	C8—C9	1.523 (3)
O1W—H12	0.843 (10)	C8—H8A	0.9700
N1—C12	1.362 (2)	C8—H8B	0.9700
N1—C1	1.428 (2)	C9—C10	1.522 (3)
N1—C13	1.476 (2)	C9—H9	0.9800
N2—C7	1.336 (2)	C10—C11	1.526 (2)
N2—C8	1.463 (2)	C10—H10A	0.9700
N2—C11	1.474 (2)	C10—H10B	0.9700
C1—C6	1.400 (2)	C11—C12	1.524 (2)
C1—C2	1.404 (2)	C11—H11A	0.9800
C2—C3	1.377 (3)	C13—C14	1.456 (3)
C2—H2	0.9300	C13—H13A	0.9700
C3—C4	1.377 (3)	C13—H13B	0.9700
C3—H3A	0.9300	C14—C15	1.176 (3)
C4—C5	1.384 (3)	C15—H15	0.9300
			
C9—O3—H3	109 (2)	N2—C8—H8B	111.2
H11—O1W—H12	106 (3)	C9—C8—H8B	111.2
C12—N1—C1	124.80 (13)	H8A—C8—H8B	109.1
C12—N1—C13	116.23 (14)	O3—C9—C10	110.79 (16)
C1—N1—C13	118.96 (14)	O3—C9—C8	113.19 (16)
C7—N2—C8	122.10 (15)	C10—C9—C8	103.21 (15)
C7—N2—C11	125.15 (13)	O3—C9—H9	109.8
C8—N2—C11	111.97 (14)	C10—C9—H9	109.8
C6—C1—C2	118.58 (15)	C8—C9—H9	109.8
C6—C1—N1	123.42 (14)	C9—C10—C11	103.99 (14)
C2—C1—N1	117.90 (14)	C9—C10—H10A	111.0
C3—C2—C1	120.83 (17)	C11—C10—H10A	111.0
C3—C2—H2	119.6	C9—C10—H10B	111.0
C1—C2—H2	119.6	C11—C10—H10B	111.0
C4—C3—C2	120.81 (17)	H10A—C10—H10B	109.0
C4—C3—H3A	119.6	N2—C11—C12	107.39 (14)
C2—C3—H3A	119.6	N2—C11—C10	103.60 (12)
C3—C4—C5	119.09 (19)	C12—C11—C10	113.32 (15)
C3—C4—H4	120.5	N2—C11—H11A	110.8
C5—C4—H4	120.5	C12—C11—H11A	110.8
C4—C5—C6	121.35 (17)	C10—C11—H11A	110.8
C4—C5—H5	119.3	O1—C12—N1	122.04 (15)
C6—C5—H5	119.3	O1—C12—C11	122.86 (16)
C5—C6—C1	119.23 (15)	N1—C12—C11	115.08 (14)
C5—C6—C7	114.86 (15)	C14—C13—N1	112.68 (14)
C1—C6—C7	125.91 (15)	C14—C13—H13A	109.1
O2—C7—N2	122.21 (15)	N1—C13—H13A	109.1
O2—C7—C6	120.45 (16)	C14—C13—H13B	109.1
N2—C7—C6	117.27 (15)	N1—C13—H13B	109.1
N2—C8—C9	102.88 (14)	H13A—C13—H13B	107.8
N2—C8—H8A	111.2	C15—C14—C13	177.6 (2)
C9—C8—H8A	111.2	C14—C15—H15	180.0
			
C12—N1—C1—C6	−47.5 (2)	C7—N2—C8—C9	171.01 (16)
C13—N1—C1—C6	132.32 (17)	C11—N2—C8—C9	−18.65 (19)
C12—N1—C1—C2	136.14 (18)	N2—C8—C9—O3	154.01 (16)
C13—N1—C1—C2	−44.0 (2)	N2—C8—C9—C10	34.20 (19)
C6—C1—C2—C3	−2.8 (3)	O3—C9—C10—C11	−159.11 (16)
N1—C1—C2—C3	173.70 (18)	C8—C9—C10—C11	−37.66 (19)
C1—C2—C3—C4	0.0 (3)	C7—N2—C11—C12	−74.4 (2)
C2—C3—C4—C5	1.8 (3)	C8—N2—C11—C12	115.58 (16)
C3—C4—C5—C6	−0.6 (3)	C7—N2—C11—C10	165.40 (18)
C4—C5—C6—C1	−2.2 (3)	C8—N2—C11—C10	−4.59 (18)
C4—C5—C6—C7	177.42 (19)	C9—C10—C11—N2	26.07 (18)
C2—C1—C6—C5	3.9 (2)	C9—C10—C11—C12	−89.97 (17)
N1—C1—C6—C5	−172.45 (16)	C1—N1—C12—O1	−179.99 (17)
C2—C1—C6—C7	−175.72 (16)	C13—N1—C12—O1	0.2 (2)
N1—C1—C6—C7	8.0 (3)	C1—N1—C12—C11	1.6 (2)
C8—N2—C7—O2	−1.4 (3)	C13—N1—C12—C11	−178.26 (14)
C11—N2—C7—O2	−170.47 (17)	N2—C11—C12—O1	−109.22 (19)
C8—N2—C7—C6	175.64 (16)	C10—C11—C12—O1	4.6 (2)
C11—N2—C7—C6	6.6 (3)	N2—C11—C12—N1	69.19 (18)
C5—C6—C7—O2	30.1 (3)	C10—C11—C12—N1	−177.03 (14)
C1—C6—C7—O2	−150.25 (19)	C12—N1—C13—C14	81.9 (2)
C5—C6—C7—N2	−146.98 (17)	C1—N1—C13—C14	−97.99 (19)
C1—C6—C7—N2	32.6 (3)	N1—C13—C14—C15	−158 (6)

### Hydrogen-bond geometry (Å, °)

**Table:**

*D*—H···*A*	*D*—H	H···*A*	*D*···*A*	*D*—H···*A*
O3—H3···O1w	0.84 (1)	1.85 (1)	2.686 (2)	177 (3)
O1w—H11···O2^i^	0.84 (1)	1.92 (1)	2.7485 (19)	172 (4)
O1w—H12···O3^ii^	0.84 (1)	1.92 (1)	2.767 (2)	177 (3)
C15—H15···O1^iii^	0.93	2.29	3.166 (3)	157

Symmetry codes: (i) −*x*+1, *y*−1/2, −*z*; (ii) −*x*+1, *y*+1/2, −*z*; (iii) −*x*+1, *y*+1/2, −*z*+1.
